# Adipose most abundant 2 protein is a predictive marker for cisplatin sensitivity in cancers

**DOI:** 10.1038/s41598-021-85498-7

**Published:** 2021-03-18

**Authors:** Kenya Kamimura, Takeshi Suda, Yasuo Fukuhara, Shujiro Okuda, Yu Watanabe, Takeshi Yokoo, Akihiko Osaki, Nobuo Waguri, Toru Ishikawa, Toshihiro Sato, Yutaka Aoyagi, Masaaki Takamura, Toshifumi Wakai, Shuji Terai

**Affiliations:** 1 Division of Gastroenterology and Hepatology, Graduate School of Medical and Dental Sciences, Niigata University, 1-757 Asahimachi–dori, Chuo–ku, Niigata, Niigata 951-8510 Japan; 2 Department of General Medicine, Niigata University School of Medicine, 1-757, Asahimachido-ri, Chuo-ku, Niigata, Niigata 951-8510 Japan; 3 Department of Gastroenterology and Hepatology, Uonuma Institute of Community Medicine Niigata University Hospital, Minamiuonuma, Niigata 949-7302 Japan; 4 Division of Bioinformatics, Graduate School of Medical and Dental Sciences, Niigata University, Niigata, Niigata 951-8510 Japan; 5 Department of Gastroenterology and Hepatology, Niigata City General Hospital, Niigata, Niigata 950-1197 Japan; 6 Department of Gastroenterology and Hepatology, Saiseikai Niigata Hospital, Niigata, Niigata 950-1104 Japan; 7 Department of Gastroenterology, Kashiwazaki General Hospital and Medical Center, Kashiwazaki, Niigata 945-8535 Japan; 8 Department of Gastroenterology and Hepatology, Niigata Medical Center, Niigata, Niigata 950-2022 Japan; 9 Division of Digestive and General Surgery, Graduate School of Medical and Dental Sciences, Niigata University, Niigata, Niigata 951-8510 Japan

**Keywords:** Cancer, Biomarkers, Gastroenterology

## Abstract

Cisplatin (CDDP) is one of the chemotherapeutic drugs being used to treat various cancers. Although effective in many cases, as high doses of CDDP cause cytotoxic effects that may worsen patients’ condition, therefore, a marker of sensitivity to CDDP is necessary to enhance the safety and efficiency of CDDP administration. This study focused on adipose most abundant 2 (APM2) to examine its potential as a marker of CDDP sensitivity. The relationship of APM2 expression with the mechanisms of CDDP resistance was examined in vitro and in vivo using hepatocellular carcinoma (HCC) cells, tissues and serum of HCC patients (n = 71) treated initially with intrahepatic arterial infusion of CDDP followed by surgical resection. The predictability of serum APM2 for CDDP sensitivity was assessed in additional 54 HCC patients and 14 gastric cancer (GC) patients. APM2 expression in CDDP-resistant HCC was significantly higher both in serum and the tissue. Bioinformatic analyses and histological analyses demonstrated upregulation of ERCC6L (DNA excision repair protein ERCC6-like) by APM2, which accounts for the degree of APM2 expression. The serum APM2 level and chemosensitivity for CDDP were assessed and cut-off value of serum APM2 for predicting the sensitivity to CDDP was determined to be 18.7 µg/mL. The value was assessed in HCC (n = 54) and GC (n = 14) patients for its predictability of CDDP sensitivity, resulted in predictive value of 77.3% and 100%, respectively. Our study demonstrated that APM2 expression is related to CDDP sensitivity and serum APM2 can be an effective biomarker of HCC and GC for determining the sensitivity to CDDP.

Trial registration: This study was registered with the University Hospital Medical Information Network Clinical Trials Registry (UMIN000028487).

## Introduction

Cisplatin (CDDP) is one of the chemotherapeutic drugs used to treat various cancers, including gastrointestinal and hepatobiliary cancers. Among them, liver cancer is one of the leading causes of cancer-related deaths worldwide^1,2^, and hepatocellular carcinoma (HCC) comprises > 90% of the primary liver cancer patients. For HCC, surgery, ablation, chemoembolization, transarterial chemotherapy, and molecularly targeted agents have been utilized, depending on the remaining hepatic function^3,4^; CDDP is one of the chemotherapeutic drugs administered for HCC treatment in advanced stages. Many HCC patients respond to CDDP, which is administered via the hepatic artery; however, some HCCs are resistant to CDDP; such tumors are lethal, and administration of high doses of CDDP not only is ineffective but also causes cytotoxic effects that may worsen patients’ condition^5^. Thus, a marker of sensitivity to CDDP is necessary for making appropriate therapeutic decisions.

In this study, we focused on the factor adipose most abundant 2 (APM2) to examine its potential as a marker of CDDP sensitivity. APM2 was discovered as the second most abundant transcript in adipose tissue, after adiponectin (APM1); it is located on chromosome 19q23.3, expressing 7.8 kDa of protein^6^; it has been reported to be expressed in a variety of other tissues, including liver, adipose, and kidney tissues^7^; it is known to promote proliferation, inhibit apoptosis, and enhance glucose transport^8^; it acts as a pro-adipogenesis factor in adipocytes^9^; and it is also known as c10orf119^8^.

APM2 has also been reported to be present in malignant tumors, with *APM2* gene overexpression in androgen-ablation-resistant prostate cancers^10^, breast cancers with a poor prognosis^11^, pancreatic intraepithelial neoplasms^12^, and CDDP-resistant gastric cancer (GC)^13^, and the high level of the serum concentration is related to urothelial cell carcinoma^14^. The in vitro study to overexpress APM2 in colon cancer cell lines has shown CDDP-resistant transformation of cells^15^, suggesting the potential contribution of APM2 to CDDP resistance; however, the study was based on the cell line assay using the colon cancer cell lines in vitro, and no clinical information and data have been assessed^15^. In addition, as colon cancer is intrinsically resistant to CDDP and it is not chosen for the chemotherapy in this type of cancer, no clinical relevance has been evidenced to date. More recently, the germline polymorphism of APM2 is reported to be related to ovarian cancer with poor prognoses^16^, and APM2 induces the upregulation of 34 micro-RNAs involved in the Wnt, transforming growth factor β, mitogen-activated protein kinase (MAPK), and Janus kinase/signal transducers and activators of transcription (Jak-STAT) pathways, indicating the oncogenic characteristics of APM2 protein^17^. And therefore, further study is essential to demonstrate the molecular mechanisms of APM2, conducting in vitro study and minute analyses of human samples and clinical data that utilize the tumors often treated with CDDP.

Therefore, in this study, we examined the relationship between the APM2 expression in the HCC cell lines and its mechanisms for CDDP resistance as well as its expression in the HCC and surrounding background liver tissue in the CDDP-treated HCC patients and analyzed the clinical information. In assessing the serum level of APM2 and chemosensitivity for CDDP in 71 cases, we determined the cut-off value of serum APM2 for predicting sensitivity to CDDP and tested this value in 54 CDDP-treated HCC and 14 chemotherapy-treated GC patients, including CDDP. To the best of our knowledge, this manuscript is the first to report the possible predictive value of the APM2 levels for CDDP sensitivity.

## Methods

### Ethical considerations

This basic and observational study protocol with the clinical samples was approved by the Ethics Committee and Institutional Review Board of Niigata University School of Medicine (Nos. 751-716 and G2018-0023, respectively), Niigata City General Hospital (13-010); Saiseikai Niigata Hospital (E13-02); and Kashiwazaki General Hospital and Medical Center (H25-05-21) and was registered with the University Hospital Medical Information Network Clinical Trials Registry (UMIN000028487). A written informed consent was obtained from all patients to collect the samples, and the study was conducted in accordance with the ethical guidance of the 1975 Declaration of Helsinki. All authors had access to the study data and had reviewed and approved the final manuscript.

### Cells

Human hepatoma HLE, HLF, HepG2, and Huh7 cell lines were purchased from the Japanese Collection of Research Bioresources Cell Bank (National Institutes of Biomedical Innovation, Health and Nutrition, Ibaraki, Osaka) and were cultured in Minimum Essential Medium, comprising 10% fetal bovine serum and 100 U/mL of penicillin and streptomycin. The APM2 complementary DNA was cloned in pCMV6-Entry Tagged Cloning Vector (OriGene Technologies, Inc., Rockville, MD). Either mock or APM2-cloned vectors were transfected into HLE, HLF, HepG2, and Huh7 cells using FuGENE HD Transfection Reagent (Promega, Madison, WI), followed by G418 sulfate selection. From each of the four cell lines, three independent clones were isolated and then used for assay.

Expression of APM2, ERCC6L, and glyceraldehyde 3-phosphate dehydrogenase (GAPDH) in cell lines was confirmed by reverse transcription polymerase chain reaction (RT-PCR). For this analysis, the RNA Easy Mini kit (Qiagen, Valencia, CA) was used to prepare total RNA from cells, according to the protocol recommended by the manufacturer. Using SuperScript II Reverse Transcriptase (Invitrogen, Carlsbad, CA), we synthesized complementary DNA from 1 to 5 mg of total RNA with an oligo (dT) primer, and we used 1 to 2 aliquots of complementary DNA products for PCR with the following primers:

- APM2 (forward): AAAGGGAGGGCTGGGGCTGAT
- APM2 (reverse): TGGTCCACCACTTGCTGAGCT
- ERCC6L (forward): GAGCAGGCTGCTCATTACCT
- ERCC6L (reverse): CAGGCTATAGAGGAAAGCTA
- GAPDH (forward): GGTCGGAGTCAACGGATTTGGTCG
- GAPDH (reverse): CCTCCGACGCCTGCTTCACCAC

Multiplex PCR was performed similarly, whereby GAPDH primers were always included as a reference. PCR products were separated by electrophoresis in 1% agarose gel and then stained with ethidium bromide for visualization.

The PCR protocol was as follows: 10 min at 95 °C, followed by 35 cycles (30 s at 95 °C, 30 s at 55 °C and 1 min at 72 °C) and then 7-min extension at 72 °C.

Expression of APM2, ERCC6L, and β-actin protein was confirmed by Western blotting. For this analysis, culture cells were suspended in phosphate-buffered saline and mixed with an equal volume of lysis buffer, 0.125 M tris–HCl (pH 6.8), 10% sucrose, 10% sodium dodecyl sulfate (SDS), 10% 2-mercaptoethanol, and 0.004% bromophenol blue. The extract was subjected to 14% SDS–polyacrylamide gel electrophoresis and blotted onto Hybond membranes (GE Healthcare Life Sciences, Pittsburgh). We used the following antibodies:

- Rabbit anti-APM2 antibody (ab79579; Abcam, Cambridge, UK)
- Rabbit anti-ERCC6L antibody (ab197925; Abcam)
- Rabbit anti-β-actin antibody (ab8227; Abcam)
- Anti-rabbit immunoglobulin G horseradish peroxidase (NA934-1ML; GE Healthcare Life Sciences, Pittsburgh)

Protein bands were visualized using the ECL plus Western Blotting Detection System (GE Healthcare Life Sciences, Pittsburgh).

### Cell growth assay

Cells were plated in 96-well tissue culture dishes, 2 × 10^4^ cells per well, in 100 µL of the medium. Some were treated with CDDP or epirubicin with the doses determined based on the previously reported studies^18,19^. Water-soluble tetrazolium salt (WST) reagents were added to the cells at the indicated times after the treatment; then the cells were counted using the Premix WST-1 Cell Proliferation Assay System (Takara Inc., Kyoto, Japan).

### Expression of APM2 in vivo and clinical course

Computed tomographic studies of HCC after the CDDP infusion were performed 4 to 6 weeks after chemotherapy as a standard follow-up of the therapeutic effect. The tissue samples for the analyses were obtained from the HCC tumor cells surgically resected in Niigata University Hospital; some samples came from patients who had received CDDP before the resection, while some from those who had no therapeutic intervention. The blood samples for enzyme-linked immunosorbent assay (ELISA) were collected from the HCC patients in Niigata University Hospital, Niigata City General Hospital, Saiseikai Niigata Hospital, and Kashiwazaki General Hospital and Medical Center, before administering any chemotherapeutic agents. The blood samples from the GC patients were collected in Niigata University Hospital before the chemotherapy, including CDDP, regardless of the prior therapeutic options. The unstained slides for skin, kidney, and adipose tissues were obtained from patients who underwent biopsy for diagnosis of their diseases, all with a written informed consent. Tissues of the skin and kidneys, adipose tissue, and HCC tumor cells and the surrounding liver tissues were stained with hematoxylin and eosin and with the following immunohistochemical stains: anti-APM2 antibody (ab79579; Abcam, Cambridge, UK), anti-ERCC6L (ab197925; Abcam), Vectastain Elite ABC rabbit immunoglobulin G kit (PK-6101; Vector Laboratories, Burlingame, CA), and 3,3′-diaminobenzidine chromogen tablets (Muto Pure Chemicals, Tokyo, Japan). Supplementary Fig. S1a and S1b show the negative control staining, in which the primary antibody was omitted. Expression of APM2 and β2-microglobulin (B2M) was confirmed by RT-PCR as described earlier. The primers used for B2M expression analyses were:

- B2M (forward): GGCTATCCAGCGTACTCCAAAG
- B2M (reverse): CAACTTCAATGTCGGATGGATG

Images were captured from each tissue section randomly, and a quantitative analysis was performed using ImageJ software (version 1.6.0_20; National Institutes of Health, Bethesda, MD)^20^.

### Enzyme-linked immunosorbent assay

Blood samples were used for serum APM2 concentration analysis by ELISA with rabbit anti-APM2 antibody (ab79579; Abcam, Cambridge, UK) and Protein Detector HRP Microwell Kit, Anti-Rabbit (5110-0010; SeraCare, Milford, MA), according to the manufacturers’ instructions.

### Microarray and bioinformatic analyses

The SurePrint G3 Human Gene Expression (v2) Microarray Kit (Agilent Technologies, Inc., Santa Clara, CA) and GeneSpring GX, version 14.5.1 (Agilent Technologies, Inc.), were used in comparing the gene expression levels in mock-transfected and APM2-transfected HLE. A total of 6169 genes with more than twofold differences in expression were clustered hierarchically according to the level of gene expression. Ten groups were assessed with related gene expressions using gene ontology term analyses. The gene ontology terms were selected on the basis of Fisher’s exact test, followed by the Benjamini-Yekutieli correction method. To construct a molecular interactions network, the protein–protein association data of the Search Tool for the Retrieval of Interacting Genes/Proteins (STRING) database^21^ was imported to Cytoscape^22^ with the stringApp plugin software. STRING imports protein association knowledge from databases of physical interaction and of curated biological pathway knowledge. From the protein–protein association network, proteins with a cut-off of 0.4 edge confidence score (default value) were selected, and the molecular interaction network was constructed with the default settings in Cytoscape.

### Statistical analyses

The obtained data were analyzed using either the paired *t-*test or a one-way/two-way factor repeated-measures analysis of variance, followed by Bonferroni’s multiple comparison test. Receiver operating characteristic (ROC) curves were used to determine the cut-off value of serum APM2 concentration using Graphpad Prism 7 software (version 7.03; MDF, Tokyo). *P* ≤ 0.05 was considered statistically significant.

### Consent for publication

A written informed consent was obtained from all patients to publish the results based from the samples and images.

## Results

### Expression of APM2 in organs

**Figure 1:**
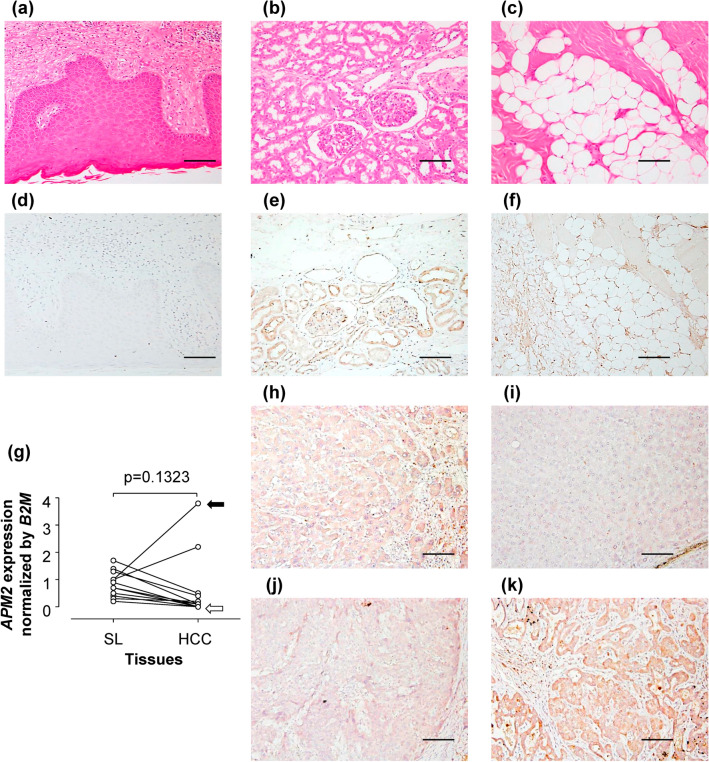
Expression of APM2 in various tissues. (**a**–**c**) Hematoxylin and eosin staining of (**a**) normal skin tissue, (**b**) normal kidney tissue, and (**c**) normal adipose tissue. (**d**–**f**) Immunohistochemical staining of APM2 of (**d**) normal skin tissue, (**e**) normal kidney tissue, and (**f**) normal adipose tissue. The scale bar represents 100 µm. (**g**–**k**) The level of APM2 in the liver and hepatocellular carcinoma (HCC) from patients who had received no chemotherapy. (**g**) Expression of *APM2* gene in the surrounding liver tissue (SL) and HCC in 12 patients. B2M, β2 microglobulin. The black arrow indicates that the case showed an increased level of APM2 gene expression in the tumor, whereas the white arrow indicates that the case showed no significant changes between the tumor and surrounding area. The significance of APM2 gene expression was examined using the paired *t*-test. (**h**–**k**) Representative immunohistochemical staining of APM2 in the liver tissues (**h**, **i**) and the HCC tissues (**j**, **k**) of each case. The scale bar represents 100 µm.

Immunohistochemical testing was performed on normal skin, kidney, muscle, and fat tissues to determine the APM2 expression in the tissue (Fig. 1a–f). The results show that APM2 was expressed in adipose, kidney, and liver tissues but not in the skin. The levels of both the *APM2* gene and the APM2 protein in the liver in the HCC tumor and noncancerous surrounding liver tissues varied in 12 patients, who had had no chemotherapy, as determined by RT-PCR and immunohistochemical testing (Fig. 1g–k). In a few patients, the changes shown in the *APM2* gene expression differed between the tumor area and surrounding areas of the resected liver tissue. For example, the black arrow in Fig. 1g indicates that the case showed an increased *APM2* gene expression in the tumor, whereas the white arrow indicates that the case showed no significant changes between the tumor and surrounding area. Supplementary Fig S1c and S1d shows the representative image of the section consisting of both normal and HCC tissues, suggesting APM2′s expression in various organs, as previously reported, and its expression level’s variability in the liver when the tumor is present.

### Effect of APM2 expression in HCC cells on the CDDP sensitivity

**Figure 2:**
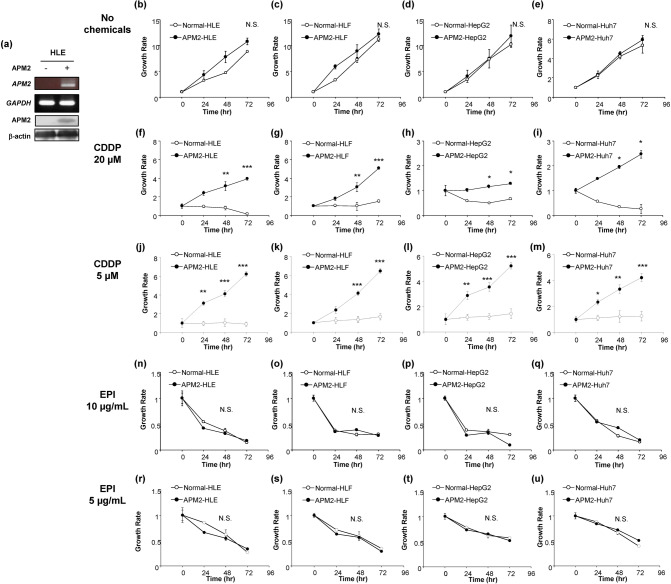
Effect of APM2 expression in HCC cells on the CDDP sensitivity. (**a**) The development of adipose most abundant 2 (APM2) overexpressing hepatocellular carcinoma (HCC) cell lines. Representative reverse transcription polymerase chain reaction (RT-PCR) of *APM2* and *glyceraldehyde 3-phosphate dehydrogenase* (*GAPDH*), and Western blotting of APM2 and β-actin in APM2 overexpressing HLE. The images were grouped from the different gels and separated with the spaces. The cell growth of HCC cell lines and the permanent clones overexpressing APM2 determined the cell growth assay. (**b**–**e**) Cell growth of cell lines with no chemicals. (**f**–**i**) Cell growth with CDDP of 20 µM and (**j**–**m**) 5 µM. (**n**–**q**) Cell growth with epirubicin (EPI) of 10 µg/mL and (**r**–**u**) 5 µg/mL. The values are expressed as mean ± standard deviation (n = 5 for each group at time points). *p < 0.05, **p < 0.01, ***p < 0.001, and NS, no statistical significance. Two-way analysis of variance followed by Bonferroni’s multiple comparisons test.

To examine the molecular function of APM2 on the liver cancer cells, we produced *APM2*-overexpressing cell lines by transfecting plasmid DNA-expressing human *APM2* into four cancer cell lines: HLE, HLF, HepG2, and Huh7. Figure 2a shows the *APM2* gene and APM2 protein expression in the transfected HLE cell line (for HLF, HepG2, and Huh7, see Supplementary Fig. S1e). A slight endogenous level of APM2 expression can be seen in the HepG2 cells, whereas the significant overexpression of APM2 was achieved by transfection. In comparison with the mock cells transfected with the plasmid with no *APM2* gene, the *APM2*-overexpressing cells showed no significant difference in growth rate under the normal culture condition determined by the cell growth assay (Fig. 2b–e). However, after administering 20 µM or 5 µM of CDDP into the medium, the cell lines with *APM2* overexpression had a significantly higher growth rate than did the mock-transfected cells, in which the cell growth was inhibited by CDDP (Fig. 2f–m). This phenomenon was not observed with other chemotherapy agents, including epirubicin, which is known to inhibit the growth of HCC cells (Fig. 2n–u), indicating that *APM2* overexpression induced resistance to CDDP in HCC cells.

### Gene expression analyses

**Figure 3:**
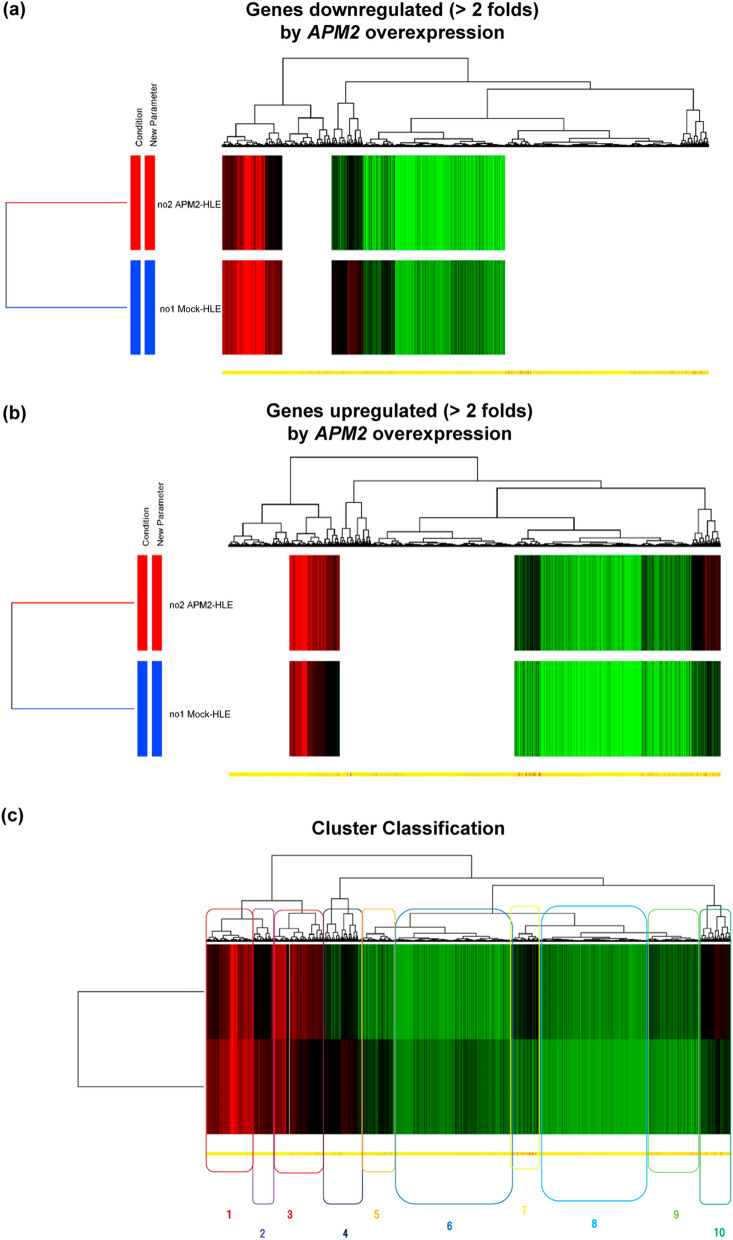

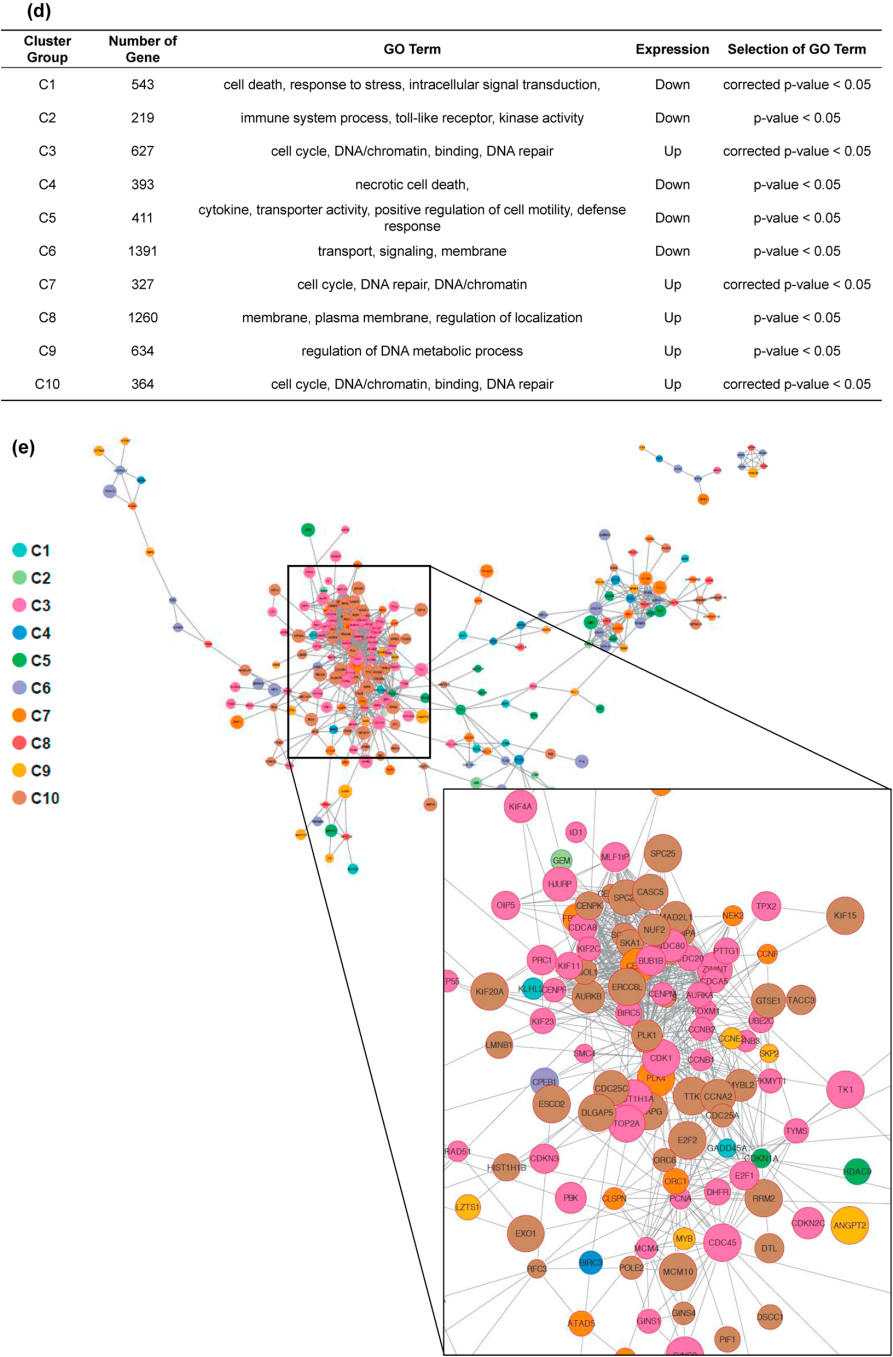
Microarray and bioinformatic analyses. Microarray analyses were performed to compare the levels of gene expression in mock-transfected HLE and APM2-transfected HLE. A total of 6169 genes with more than two-fold differences in the expression were clustered hierarchically according to level of gene expression. (**a**) Downregulated (> 2 folds) and (**b**) upregulated (> 2 folds) genes. The color represents the expression level of the gene: green represents low changes of expression, whereas red represents high changes of expression. The expression levels are continuously mapped on the color scale (**a**, 2–247.1-fold change; **b**, 2–69.2-fold change). (**c**, **d**) Ten groups were assessed with related gene expressions using gene ontology term analyses. (**e**) Gene expression network analysis based on the microarray assays using the in vitro* APM2* overexpressing cell lines (HLE). Inset focuses on the significantly related genes. APM2, adipose most abundant 2.

**Figure 4:**
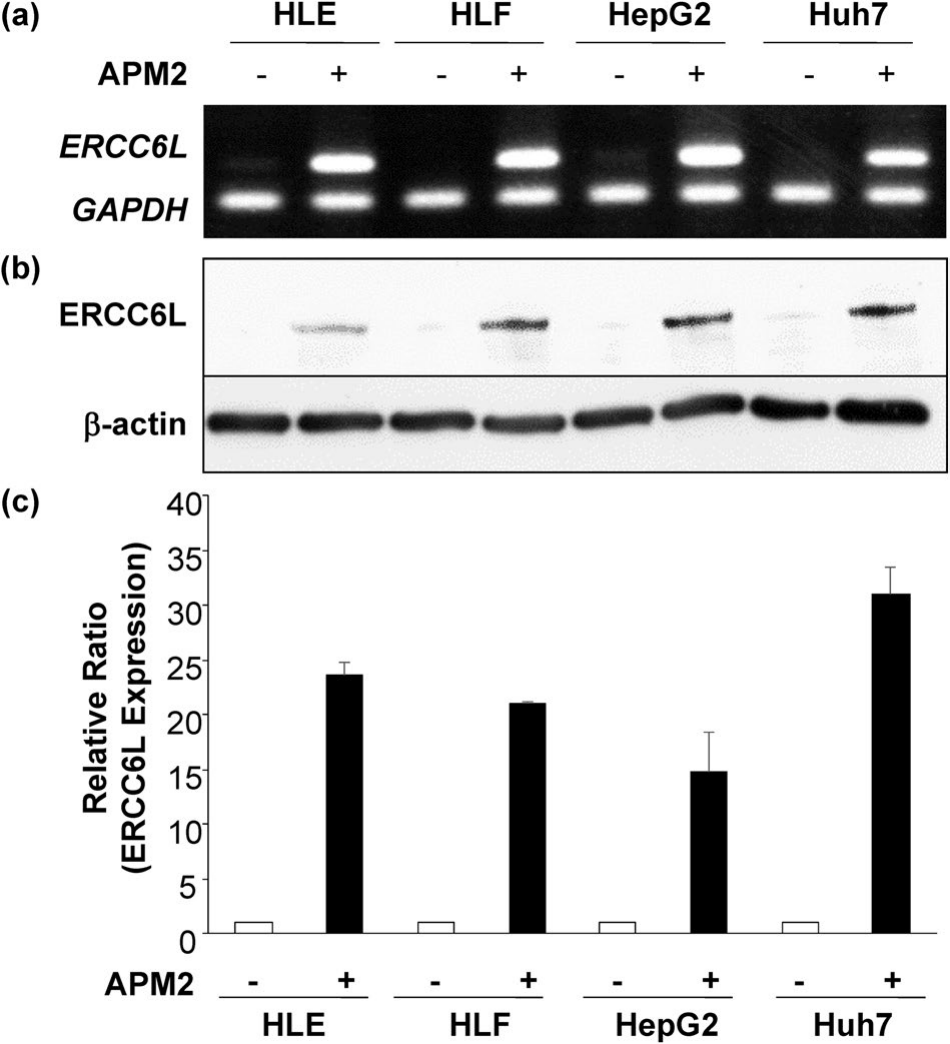
ERCC6L expression in the HCC cell lines with APM2 overexpression. (**a**) *ERCC6L* expression in the HCC cell lines assessed by RT-PCR. (**b**) ERCC6L expression in the HCC cell lines assessed by Western blotting. The images were grouped from the different gels and separated with the spaces. (**c**) A quantitative analysis of ERCC6L protein expression in the cell lines. Relative band ratio using the band intensities of β-actin as references. Results are expressed as mean, and error bars are expressed as standard deviations of three independent experiments. APM2, adipose most abundant 2.

To determine the molecular mechanism of chemoresistance to CDDP, the gene expression in mock-transfected HLE and APM2-transfected HLE were compared using DNA microarray analyses. Figure 3a, b shows the representative data from the HLE cell lines. The analysis of gene ontology terms after the hierarchical clustering of genes showed gene differences in terms of cell death, cell cycle, DNA repair, DNA/chromatin, transporter, kinase activity, and other characteristics, which were grouped as C1 to C10 (Fig. 3c, d). Enrichment analyses were performed on the basis of these gene expressions; those that were more than fivefold higher were used in developing the molecular interactions network (see Materials and Methods section) (Fig. 3e). Of importance was the network’s significant relation to the C3 and C10 groups, determined by the clustering analysis, including cell cycle, DNA/chromatin, binding, and DNA repair (corrected *p* < 0.05) (Fig. 3e). These groups include genes of E2F2 (23.4-fold change), BRCA1-interacting protein 1 (27.5-fold change), Thymosin Beta gene (69.2-fold change), etc., which are related to the cell growth. Among these genes, we focused on *ERCC6L*, with a 23.466-fold higher expression in *APM2*-overexpressed cells, because it has been reported to be related to DNA repair and chromosomal instability^23^, cell growth^24–27^, and cell cycle^28^. *ERCC6L* expression was significantly higher in all four *APM2*-overexpressed cell lines, as shown in RT-PCR (Fig. 4a) and Western blotting (Fig. 4b). A significantly high relative ratio of ERCC6L protein expression in *APM2*-overexpressed cells to that in mock-transfected cells was noted (Fig. 4c).

### APM2 expression in human HCC and CDDP sensitivity

**Figure 5:**
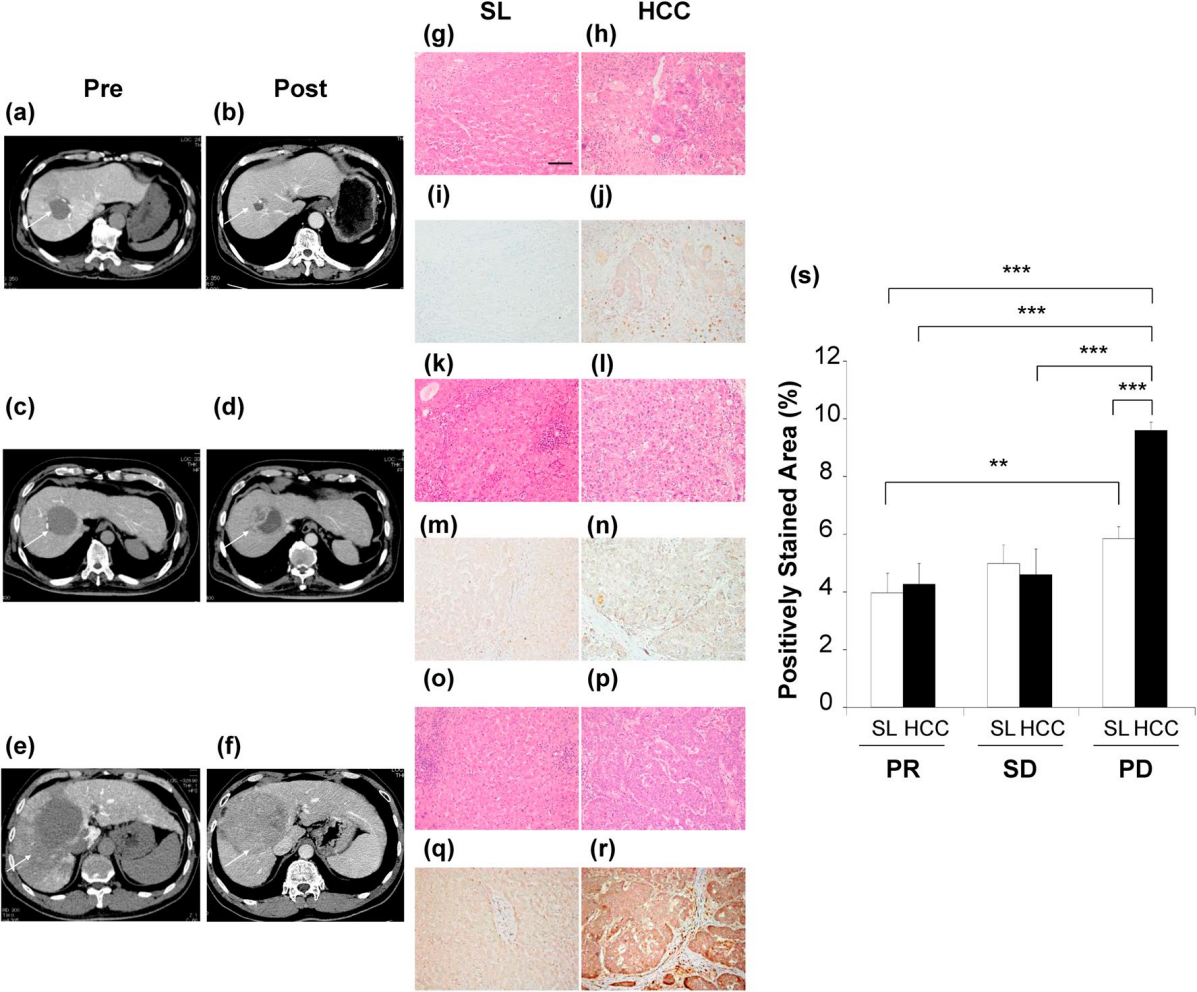

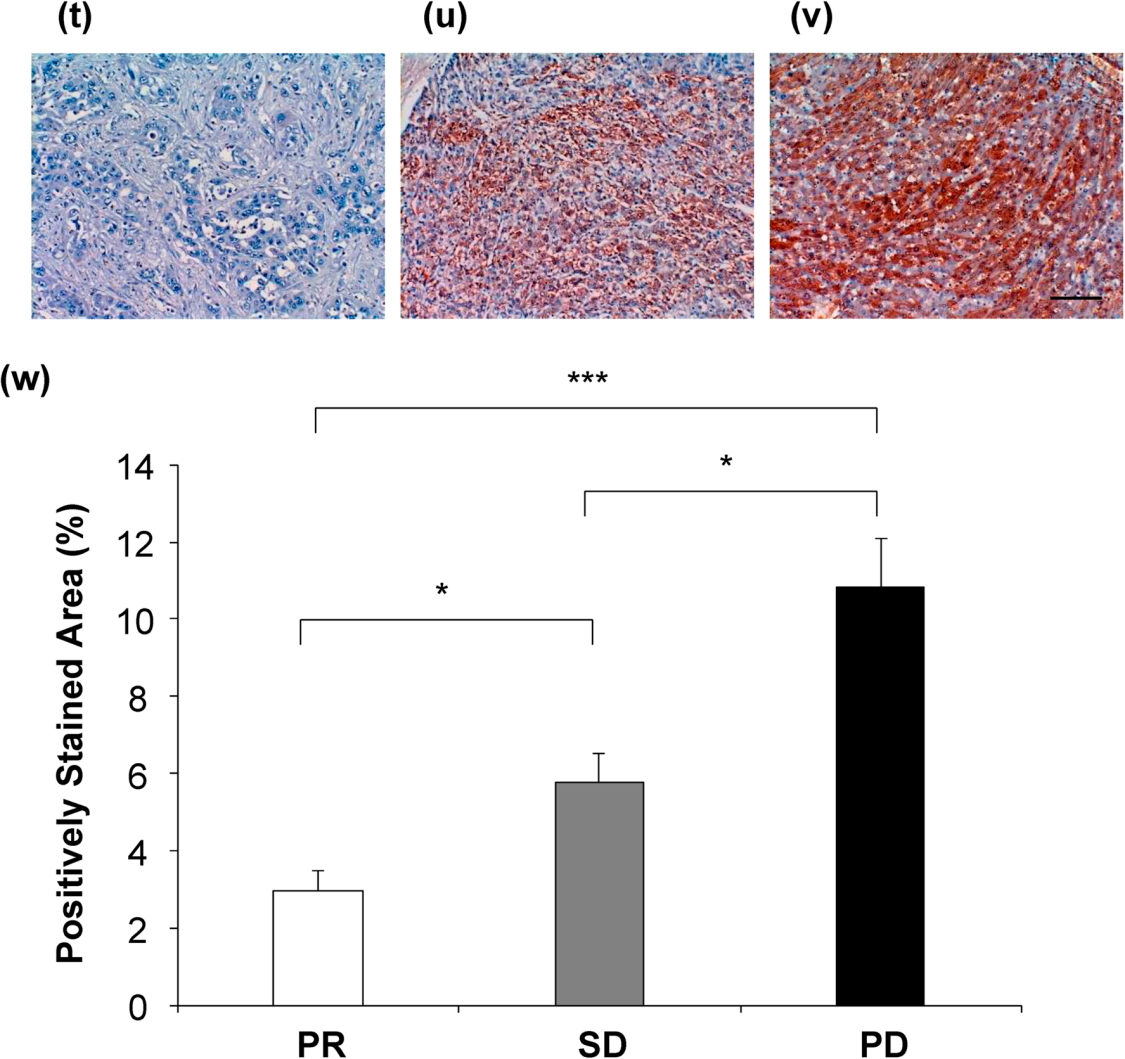
The APM2 expression in the liver tissue and chemosensitivity to CDDP. The representative computed tomography images before and after the transcatheter arterial infusion of cisplatin (CDDP) to the hepatocellular carcinoma (HCC) resulted in the partial response (PR), stable disease (SD) and progressive disease (PD). White arrows indicate the tumors. (**a**, **b**) PR case, (**c**, **d**) SD case, (**e**, **f**) PD case. White arrows indicate the tumor. (**g**–**r**) Representative histological analyses. Hematoxylin and eosin staining of surrounding liver tissue (SL) and HCC in PR case (**g**, **h**), SD cases (**k**, **l**) and PD case (**o**, **p**). Immunohistochemical staining of APM2 of SL and HCC in PR case (**i**, **j**), SD cases (**m**, **n**) and PD case (**q**, **r**). The scale bar represents 100 µm. (**s**) A quantitative analysis of positively stained area in SL and HCC tissues with APM2. For quantitative measurements, 3 representative images from each patient in 3 groups (PR, n = 4, SD, n = 6, PD, n = 3) were captured at a final magnification of × 200. Quantification was performed measuring the integrated density in pixels using the ImageJ software (version 1.6.0_20, National Institutes of Health, USA). The APM2 stained/negative control ratio of the integrated density in pixels was calculated as the relative integrated density. The values represent means ± standard deviations. APM2, adipose most abundant 2. **p < 0.01, ***p < 0.001, One-way analysis of variance followed by Bonferroni’s multiple comparison test. (**t**–**v**) The ERCC6L expression in the liver tissue and chemosensitivity to CDDP. The representative immunohistochemical staining of ERCC6L in the hepatocellular carcinoma (HCC) showed partial response (PR), stable disease (SD) and progressive disease (PD). (**t**) PR case, (**u**) SD case, (**v**) PD case. The scale bar represents 100 µm. (**w**) A quantitative analysis of positively stained cells with ERCC6L in 3 groups (PR, n = 4, SD, n = 6, PD, n = 3). Quantification was performed measuring the integrated density in pixels using the ImageJ software (version 1.6.0_20, National Institutes of Health, USA). The ERCC6L stained/negative control ratio of the integrated density in pixels was calculated as the relative integrated density. The values represent means ± standard deviations. *p < 0.05, ***p < 0.001, One-way analysis of variance followed by Bonferroni’s multiple comparison test.

To examine the relationship between the APM2 levels in HCC and in surrounding liver tissues, as well as sensitivity to CDDP treatment, the liver tissues of patients treated first with hepatic arterial infusion of CDDP and then with surgical resection were subjected to immunohistochemical analyses. On the basis of the clinical course, the expression of APM2 was assessed in patients with partial response (PR group) to treatment, those with a stable disease (SD group), and those with a progressive disease (PD group) after CDDP administration. Representative computed tomographic images are shown in Fig. 5a–f.

Positively stained areas were quantitatively analyzed in several cases in each group (4, 6, and 3 in the PR, SD, and PD groups, respectively) (Fig. 5g–s), the results of which indicate a significantly higher APM2 expression in the tumor tissue among patients in the PD group than in those of the PR and SD groups (*p* < 0.001) (Fig. 5s). In addition, a significantly higher level in the surrounding tissue of patients in the PD group than in that of patients in the PR group (*p* < 0.01) was noted. To determine whether ERCC6L expression was correlated with both APM2 expression and CDDP chemosensitivity in human tissues, the tumors were subjected to immunohistochemical analyses (Fig. 5t–v). The expression of ERCC6L was significantly higher in PD tumors, which showed a poorer response to CDDP (Fig. 5v) than in PR (Fig. 5t) (*p* < 0.001) or SD tumors (Fig. 5u) (*p* < 0.05), and it was significantly higher in the SD group than in the PR group (*p* < 0.05) (Fig. 5w), suggesting that the APM2 expression may contribute to ERCC6L upregulation in the HCC tissue and may be related to the chemosensitivity to CDDP.

### Serum APM2 concentration and sensitivity of HCC to CDDP

**Figure 6:**
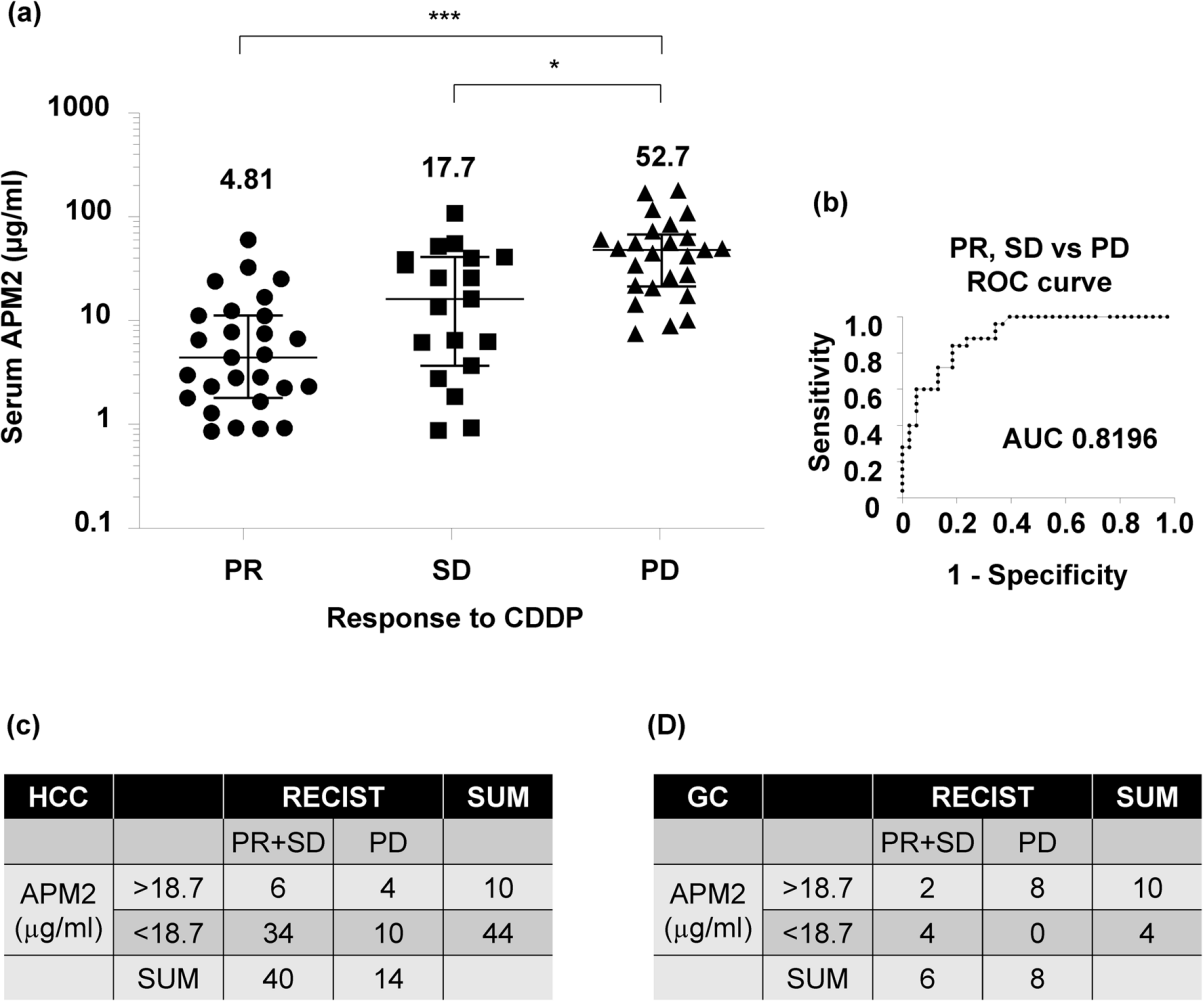
Serum concentration of APM2 and chemosensitivity to CDDP in the Patients with HCC or GC. (**a**) enzyme-linked immunosorbent assay (ELISA) was performed on serum collected from 71 patients described before administration of cisplatin (CDDP). Partial response (PR), n = 27; stable disease (SD), n = 19; and progressive disease (PD), n = 25. The values represent mean ± standard deviations. *p < 0.05, ***p < 0.001, One-way analysis of variance followed by Bonferroni’s multiple comparison test. (**b**) Receiver operating characteristic (ROC) curves are used to determine a cut-off value for “PR & SD” vs “PD”. (**c**) A prediction of CDDP chemosensitivity for HCC based on the cut-off value of 18.7 µg/mL. (**d**) A prediction of CDDP chemosensitivity for gastric cancer based on the cut-off value of 18.7 µg/mL. The anti-tumor responses were determined after the first evaluation based on the RECIST or endoscopic findings. *APM2* adipose most abundant 2, *RECIST* response evaluation criteria in solid tumors; *GC* gastric cancer.

To determine serum APM2 concentration as a potential biomarker of CDDP sensitivity, as it is secreted into the blood stream, the APM2 serum level was tested with ELISA in 71 HCC patients who were treated with CDDP intra-arterial infusion (Table 1). The concentration of serum APM2 and the response to CDDP administration were assessed according to the response evaluation criteria in solid tumors classification^29^. The mean serum concentrations were 4.81 ± 9.2 µg/mL in the PR group (n = 27), 17.7 ± 23.6 µg/mL in the SD group (n = 19), and 52.7 ± 10.2 µg/mL in the PD group (n = 25); the serum concentration was thus significantly higher in the PD group than in the SD group (*p* < 0.05) and the PR group (*p* < 0.001) (Fig. 6a). The ROC curves to determine a cut-off value for the PR and SD groups, in comparison with a cut-off for the PD group, revealed that the area under the curve was 0.8196 (95% confidence interval: 0.72 to 0.92; *p* < 0.0001). The serum APM2 concentration’s cut-off value was determined as 18.7 µg/mL, with a sensitivity and specificity of 84.0% and 71.7%, respectively (Fig. 6b).

**Table 1:** Patient characteristics.

Group	PR	SD	PD	Kruskal–Wallis test
	n = 27	n = 19	n = 25	*P* value
Characteristics				
Age (years)				0.95
Median	72.0	70.0	70.0	
Range	62–80	55–78	49–82	
Gender				0.35
Female	6	7	7	
Male	21	12	18	
Etiology				0.89
HBV infection	7	4	4	
HCV infection	14	10	15	
Nonalcoholic steatohepatitis	3	3	2	
Alcohol	3	2	3	
Autoimmune hepatitis	0	0	0	
Primary biliary cirrhosis	0	0	1	
Cirrhosis				
Yes/no	24/3	16/3	25/0	
Child–pugh grade				0.43
A	22	14	23	
B	2	2	1	
C	0	0	1	
AFP (ng/ml)				0.12
Median	40.6	15.2	21.2	
Range	3.5–6390	2.1–9146	2.0–3336	

The values represent median and range. Kruskal–Wallis test was followed by Dunn’s multiple comparison test.

*HBV* hepatitis B virus, *HCV* hepatitis C virus, *PD* progressive disease, *PR* partial response, *SD* stable disease.

To examine whether the cut-off value of serum APM2 concentration could help predict the anti-tumor effect of CDDP, we assessed the serum APM2 levels in 54 HCC patients treated with CDDP (Fig. 6c) as a prospective observational study. These patients included 40, who were determined to have PR or SD, and 14 to have PD. The serum was collected before the treatment, and the assessment of the anti-tumor effect was conducted within 1 month after CDDP treatment. The negative predictive value (predicting that HCC was not PD) at a serum APM2 level of < 18.7 µg/mL was 77.3% (Fig. 6c). In addition to the HCC patients, the same assessment was conducted for GC patients treated with chemotherapy, including CDDP (n = 14), with a negative predictive value of 100% (Fig. 6d).

## Discussion

Our study suggested that APM2 expression is related to CDDP sensitivity in HCC cells and serum APM2 can be an effective biomarker of HCC and GC for determining the sensitivity to CDDP. The serum APM2 level was correlated with ERCC6L expression levels, and the cut-off value of 18.7 µg/mL enabled the effective prediction in the patients of HCC and CDDP-treated GC, according to the prospective study results. APM2 is known to be highly expressed in adipose, kidney, gastrointestinal tract, and prostate tissues, among others^30^, and is localized primarily within the nucleus^9^. As a nuclear factor, it helps to modulate various transcription factors^9^ and has been reported to be related to the malignancy potential of cancers^8–17^. However, no reports have clearly shown the relation between APM2 and malignancy potential or its effect in HCC. Our results explain the correlation between APM2 expression and development of resistance to CDDP by showing the upregulation of ERCC6L in vitro and in vivo*.*

CDDP is currently used to treat head and neck, esophageal, colorectal, bladder, lung, ovarian, and testicular cancers, as well as HCC^31^, and it binds to DNA and inhibits its replication by generating unrepairable DNA lesions that arrest cell proliferation and cause apoptosis^31^. CDDP resistance can be caused by the following: (1) alterations in the binding of CDDP to the targets; (2) alteration of the signaling pathways by CDDP, such as the increase in DNA repair, tolerance of DNA lesions, and activation of the cell cycle; (3) alterations of signaling pathways triggered by the molecular damages, such as inhibition of cell death; and (4) alterations of molecular pathways not directly related to CDDP signaling pathways^31,32^. Our microarray and bioinformatic analyses (Fig. 3) demonstrated the listed second and third causes, and the enrichment analyses indicated the significant relation between ERCC6L and APM2 overexpression and CDDP resistance.

Among the various excision repair pathways related to CDDP resistance^33–37^, ERCC proteins and polo-like kinase 1^33^ have been reported to be involved. ERCC6L is a DNA helicase and a protein of the SNF2 family of DNA translocases that binds to ultra-fine DNA bridges in mitosis; together with topoisomerase II, it prevents chromosome mis-segregation and interacts with polo-like kinase 1; thus, it is known as polo-like kinase 1-interacting checkpoint helicase^28^. In addition, ERCC6L promotes clinical progression of tumors and worsens the prognosis of human colorectal cancer^24^ and chromophobe renal cell carcinoma^25^ by modulating MAPK signaling^26^ and preserving chromosomal integrity in rapidly proliferating cells, including tumor cells^23^. And the suppression of ERCC6L in breast cancer has also been reported to inhibit tumor cell growth^27^. Therefore, our results suggest that the ERCC6L activation via APM2 expression helps HCC become CDDP resistant by promoting cell growth under DNA damage by CDDP.

According to our results, determining the serum APM2 concentration could help predict chemosensitivity to CDDP. APM2 partly contributes to ERCC6L expression in the tumors as a nuclear factor^9^ and enhances gene expressions of cell cycle and DNA/chromatin DNA repairs^38^, including ERCC6L; therefore, it can serve as a biomarker for the predictor of sensitivity to CDDP. The use of this biomarker in clinical practice to determine chemosensitivity will enhance the safety and efficiency of CDDP administration. This study thus represents a milestone for detecting CDDP sensitivity, and further study will help modify APM2 expression, which could contribute to the chemosensitization of the tumor.

The limitations of our study are as follows: lack of a molecular-based analysis of the direct link between APM2 and ERCC6L and molecular function of ERCC6L, limited sample size, and limited number of cell lines used. Therefore, further basic research, focusing on the molecular mechanisms of APM2 to determine how it affects ERCC6L and other related molecules, is needed. In addition, as microarray and bioinformatic analyses indicate the potential correlation of various genes, further assessment using human samples is essential in determining the biological role of APM2. Furthermore, the usefulness of APM2 as a biomarker in predicting the CDDP sensitivity in other cancers, including GC for which the number was small in our study, should be examined clinically.

## Conclusions

Our results demonstrated the significant relationship between the high level of APM2 expression in serum and HCC and surrounding liver tissue and CDDP resistance. This mechanism is based on the upregulation of ERCC6L protein by APM2 to promote cell growth, as also evidenced by the bioinformatic analyses using the various HCC cell lines and histological analyses of the collected liver samples. In addition, to the best of our knowledge, this is the first report to demonstrate that the serum level of APM2 can be the predictor of the CDDP chemosensitivity.

## Supplementary Information

Supplementary Information 1.

Supplementary Figure S1 Legend.

## Abbreviations

APM2: Adipose most abundant 2
CDDP: Cisplatin
HCC: Hepatocellular carcinoma
GC: Gastric cancer
ERCC6L: DNA excision repair protein ERCC6-like
GAPDH: Glyceraldehyde-3-phosphate dehydrogenase
CMV: Cytomegalovirus
PR: Partial response
SD: Stable disease
PD: Progressive disease
RECIST: Response evaluation criteria in solid tumors
SNF2: SWI/SNF catalytic subunit SNF2
MAPK: Mitogen-activated protein kinase

## References

[1] Bray F, Ferlay J, Soerjomataram I, Siegel RL, Torre LA, Jemal A (2018). Global cancer statistics 2018: GLOBOCAN estimates of incidence and mortality worldwide for 36 cancers in 185 countries. CA Cancer J. Clin..

[2] Yang JD, Hainaut P, Gores GJ, Amadou A, Plymoth A, Roberts LR (2019). A global view of hepatocellular carcinoma: trends, risk, prevention and management. Nat. Rev. Gastroenterol. Hepatol..

[3] Bruix J, Reig M, Sherman M (2016). Global cancer statistics 2018: Evidence-based diagnosis, staging, and treatment of patients with hepatocellular carcinoma. Gastroenterology.

[4] Llovet JM, Zucman-Rossi J, Pikarsky E, Sangro B, Schwartz M, Sherman M, Gores G (2016). Hepatocellular carcinoma. Nat. Rev. Dis. Primers.

[5] Osaki A, Suda T, Kamimura K, Tsuchiya A, Tamura Y, Takamura M, Igarashi M, Kawai H, Yamagiwa S, Aoyagi Y (2013). A safe and effective dose of cisplatin in hepatic arterial infusion chemotherapy for hepatocellular carcinoma. Cancer Med..

[6] Maeda K, Okubo K, Shimomura I, Funahashi T, Matsuzawa Y, Matsubara K (1996). cDNA cloning and expression of a novel adipose specific collagen-like factor, apM1 (AdiPose Most abundant Gene transcript 1). Biochem. Biophys. Res. Commun..

[7] Yanai I, Benjamin H, Shmoish M, Chalifa Caspi V, Shklar M, Ophir R, Bar Even A, Horn Saban S, Safran M, Domany E, Lancet D, Shmueli O (2005). Genome-wide midrange transcription profiles reveal expression level relationships in human tissue specification. Bioinformatics.

[8] Chen L, Zhou XG, Zhou XY, Zhu C, Ji CB, Shi CM, Qiu J, Guo XR (2013). Overexpression of C10orf116 promotes proliferation, inhibits apoptosis and enhances glucose transport in 3T3-L1 adipocytes. Mol. Med. Rep..

[9] Ni Y, Ji C, Wang B, Qiu J, Wang J, Guo X (2013). A Novel pro-adipogenesis factor abundant in adipose tissues and over-expressed in obesity acts upstream of PPARgamma and C/EBPalpha. J. Bioenerg. Biomembr..

[10] Holzbeierlein J, Lal P, LaTulippe E, Smith A, Satagopan J, Zhang L, Ryan C, Smith S, Scher H, Scardino P, Reuter V, Gerald WL (2004). Gene expression analysis of human prostate carcinoma during hormonal therapy identifies androgen-responsive genes and mechanisms of therapy resistance. Am. J. Pathol..

[11] Onda M, Emi M, Nagai H, Nagahata T, Tsumagari K, Fujimoto T, Akiyama F, Sakamoto G, Makita M, Kasumi F, Miki Y, Tanaka T, Tsunoda T, Nakamura Y (2004). Gene expression patterns as marker for 5-year postoperative prognosis of primary breast cancers. J. Cancer Res. Clin. Oncol..

[12] Prasad NB, Biankin AV, Fukushima N, Maitra A, Dhara S, Elkahloun AG, Hruban RH, Goggins M, Leach SD (2005). Gene expression profiles in pancreatic intraepithelial neoplasia reflect the effects of Hedgehog signaling on pancreatic ductal epithelial cells. Cancer Res..

[13] Kang HC, Kim IJ, Park JH, Shin Y, Ku JL, Jung MS, Yoo BC, Kim HK, Park JG (2004). Identification of genes with differential expression in acquired drug-resistant gastric cancer cells using high-density oligonucleotide microarrays. Clin. Cancer Res..

[14] Marin-Aguilera M, Mengual L, Ribal MJ, Ars E, Rios J, Gazquez C, Villavicencio H, Alcaraz A (2012). Utility of urothelial mRNA markers in blood for staging and monitoring bladder cancer. Urology.

[15] Scott BJ, Qutob S, Liu QY, Ng CE (2009). APM2 is a novel mediator of cisplatin resistance in a variety of cancer cell types regardless of p53 or MMR status. Int. J. Cancer.

[16] Braun R, Finney R, Yan C, Chen QR, Hu Y, Edmonson M, Meerzaman D, Buetow K (2013). Discovery analysis of TCGA data reveals association between germline genotype and survival in ovarian cancer patients. PLoS ONE.

[17] Qiu J, Zhou XG, Zhou XY, Zhu C, Shi CM, Ji CB, Cheng R, Li Y, Guo XR (2013). Characterization of microRNA expression profiles in 3T3-L1 adipocytes overexpressing C10orf116. Mol. Biol. Rep..

[18] Li Q, Zhu LZ, Yang RJ, Zhu X (2014). Cytotoxic activity of anticancer drugs on hepatocellular carcinoma cells in hypoxic-hyponutritional culture. Int. Surg..

[19] Wu Z, Wu J, Fang P, Kan S (2017). Puerarin increases the chemosensitivity of hepatocellular carcinoma cells. Oncol. Lett..

[20] Vrekoussis T, Chaniotis V, Navrozoglou I, Dousias V, Pavlakis K, Stathopoulos EN, Zoras O (2009). Image analysis of breast cancer immunohistochemistry-stained sections using ImageJ: An RGB-based model. Anticancer Res..

[21] Szklarczyk D, Morris JH, Cook H, Kuhn M, Wyder S, Simonovic M, Santos A, Doncheva NT, Roth A, Bork P, Jensen LJ, von Mering C (2017). The STRING database in 2017: quality-controlled protein-protein association networks, made broadly accessible. Nucleic Acids Res..

[22] Smoot ME, Ono K, Ruscheinski J, Wang PL, Ideker T (2011). Cytoscape 2.8: new features for data integration and network visualization. Bioinformatics.

[23] Albers E, Sbroggio M, Pladevall-Morera D, Bizard AH, Avram A, Gonzalez P, Martin-Gonzalez J, Hickson ID, Lopez-Contreras AJ (2018). Loss of PICH results in chromosomal instability, p53 activation, and embryonic lethality. Cell Rep..

[24] Xie Y, Yu J, Wang F, Li M, Qiu X, Liu Y, Qi J (2019). ERCC6L promotes cell growth and invasion in human colorectal cancer. Oncol. Lett..

[25] Yin X, Wang J, Zhang J (2018). Identification of biomarkers of chromophobe renal cell carcinoma by weighted gene co-expression network analysis. Cancer Cell Int..

[26] Zhang G, Yu Z, Fu S, Lv C, Dong Q, Fu C, Kong C, Zeng Y (2019). ERCC6L that is up-regulated in high grade of renal cell carcinoma enhances cell viability in vitro and promotes tumor growth in vivo potentially through modulating MAPK signalling pathway. Cancer Gene Ther..

[27] Liu J, Sun J, Zhang Q, Zeng Z (2018). shRNA knockdown of DNA helicase ERCC6L expression inhibits human breast cancer growth. Mol. Med. Rep..

[28] Nielsen CF, Huttner D, Bizard AH, Hirano S, Li TN, Palmai-Pallag T, Bjerregaard VA, Liu Y, Nigg EA, Wang LH, Hickson ID (2015). PICH promotes sister chromatid disjunction and co-operates with topoisomerase II in mitosis. Nat. Commun..

[29] Eisenhauer EA, Therasse P, Bogaerts J, Schwartz LH, Sargent D, Ford R, Dancey J, Arbuck S, Gwyther S, Mooney M, Rubinstein L, Shankar L, Dodd L, Kaplan R, Lacombe D, Verweij J (2009). New response evaluation criteria in solid tumours: revised RECIST guideline (version 1.1). Eur. J. Cancer.

[30] Fagerberg L, Hallstrom BM, Oksvold P, Kampf C, Djureinovic D, Odeberg J, Habuka M, Tahmasebpoor S, Danielsson A, Edlund K, Asplund A, Sjostedt E, Lundberg E, Szigyarto CA, Skogs M, Takanen JO, Berling H, Tegel H, Mulder J, Nilsson P, Schwenk JM, Lindskog C, Danielsson F, Mardinoglu A, Sivertsson A, von Feilitzen K, Forsberg M, Zwahlen M, Olsson I, Navani S, Huss M, Nielsen J, Ponten F, Uhlen M (2014). Analysis of the human tissue-specific expression by genome-wide integration of transcriptomics and antibody-based proteomics. Mol. Cell Proteom..

[31] Galluzzi L, Vitale I, Michels J, Brenner C, Szabadkai G, Harel-Bellan A, Castedo M, Kroemer G (2014). Systems biology of cisplatin resistance: Past, present and future. Cell Death Dis..

[32] Lu HP, Chao CC (2012). Cancer cells acquire resistance to anticancer drugs: an update. Biomed. J..

[33] Tyagi S, Bhui K, Singh R, Singh M, Raisuddin S, Shukla Y (2010). Polo-like kinase1 (Plk1) knockdown enhances cisplatin chemosensitivity via up-regulation of p73alpha in p53 mutant human epidermoid squamous carcinoma cells. Biochem. Pharmacol..

[34] Wheeler HE, Gamazon ER, Stark AL, O'Donnell PH, Gorsic LK, Huang RS, Cox NJ, Dolan ME (2013). Genome-wide meta-analysis identifies variants associated with platinating agent susceptibility across populations. Pharmacogenomics J..

[35] Stubbert LJ, Smith JM, McKay BC (2010). Decreased transcription-coupled nucleotide excision repair capacity is associated with increased p53- and MLH1-independent apoptosis in response to cisplatin. BMC Cancer.

[36] Luo Y, Fu Y, Huang R, Gao M, Liu F, Gui R, Nie X (2019). CircRNA_101505 sensitizes hepatocellular carcinoma cells to cisplatin by sponging miR-103 and promotes oxidored-nitro domain-containing protein 1 expression. Cell Death Discov..

[37] Chen S, Yang C, Sun C, Sun Y, Yang Z, Cheng S, Zhuge B (2019). miR-21-5p suppressed the sensitivity of hepatocellular carcinoma cells to cisplatin by targeting FASLG. DNA Cell Biol..

[38] Weigel C, Chaisaingmongkol J, Assenov Y, Kuhmann C, Winkler V, Santi I, Bogatyrova O, Kaucher S, Bermejo JL, Leung SY, Chan TL, Lasitschka F, Bohrer MH, Marx A, Haußen RH, Herold-Mende C, Dyckhoff G, Boukamp P, Delank KW, Hörmann K, Lippert BM, Baier G, Dietz A, Oakes CC, Plass C, Becher H, Schmezer P, Ramroth H, Popanda O (2019). DNA methylation at an enhancer of the three prime repair exonuclease 2 gene (TREX2) is linked to gene expression and survival in laryngeal cancer. Clin. Epigenet..

