# Supplementary material for: Adipose most abundant 2 protein is a predictive marker for cisplatin sensitivity in cancers
Source: Sci Rep. 2021 Mar 18;11:6255. doi: 10.1038/s41598-021-85498-7 (PMC7973578; doi:10.1038/s41598-021-85498-7)

Supplementary Information

Adipose Most Abundant 2 Protein is a Predictive Marker for Cisplatin Sensitivity in Cancers

Kenya Kamimura,[1]* Takeshi Suda,[2] Yasuo Fukuhara,[1] Shujiro Okuda,[3] Yu Watanabe,[3] Takeshi Yokoo,[1] Akihiko Osaki,[4] Nobuo Waguri,[4] Toru Ishikawa,[5] Toshihiro Sato,[6] Yutaka Aoyagi,[7] Masaaki Takamura,[1] Toshifumi Wakai,[8] and Shuji Terai[1]

[1]Division of Gastroenterology and Hepatology, Graduate School of Medical and Dental Sciences, Niigata University, Niigata, Niigata, 951-8510, Japan

[2]Department of Gastroenterology and Hepatology, Uonuma Institute of Community Medicine Niigata University Hospital, Minamiuonuma, Niigata, 949-7302, Japan

[3]Division of Bioinformatics, Graduate School of Medical and Dental Sciences, Niigata University, Niigata, Niigata, 951-8510, Japan

[4]Department of Gastroenterology and Hepatology, Niigata City General Hospital, Niigata, Niigata, 950-1197, Japan

[5]Department of Gastroenterology and Hepatology, Saiseikai Niigata Hospital, Niigata, Niigata, 950-1104, Japan

[6]Department of Gastroenterology, Kashiwazaki General Hospital and Medical Center, Kashiwazaki, Niigata 945-8535, Japan

[7]Department of Gastroenterology and Hepatology, Niigata Medical Center, Niigata, Niigata, 950-2022, Japan

[8]Division of Digestive and General Surgery, Graduate School of Medical and Dental Sciences, Niigata University, Niigata, Niigata, 951-8510, Japan

*Correspondence should be addressed to: Kenya Kamimura, M.D., Ph.D.

Division of Gastroenterology and Hepatology

Graduate School of Medical and Dental Sciences

Niigata University

1–757 Asahimachi–dori

Chuo–ku, Niigata, Niigata, 951-8510

Tel: +81 (25) 227–2207

Fax: +81 (25) 227–0776

E–mail*:* kenya–k@med.niigata–u.ac.jp

Short running title: ***APM2 is a novel marker for CDDP sensitivity***

Supplementary Figure S1

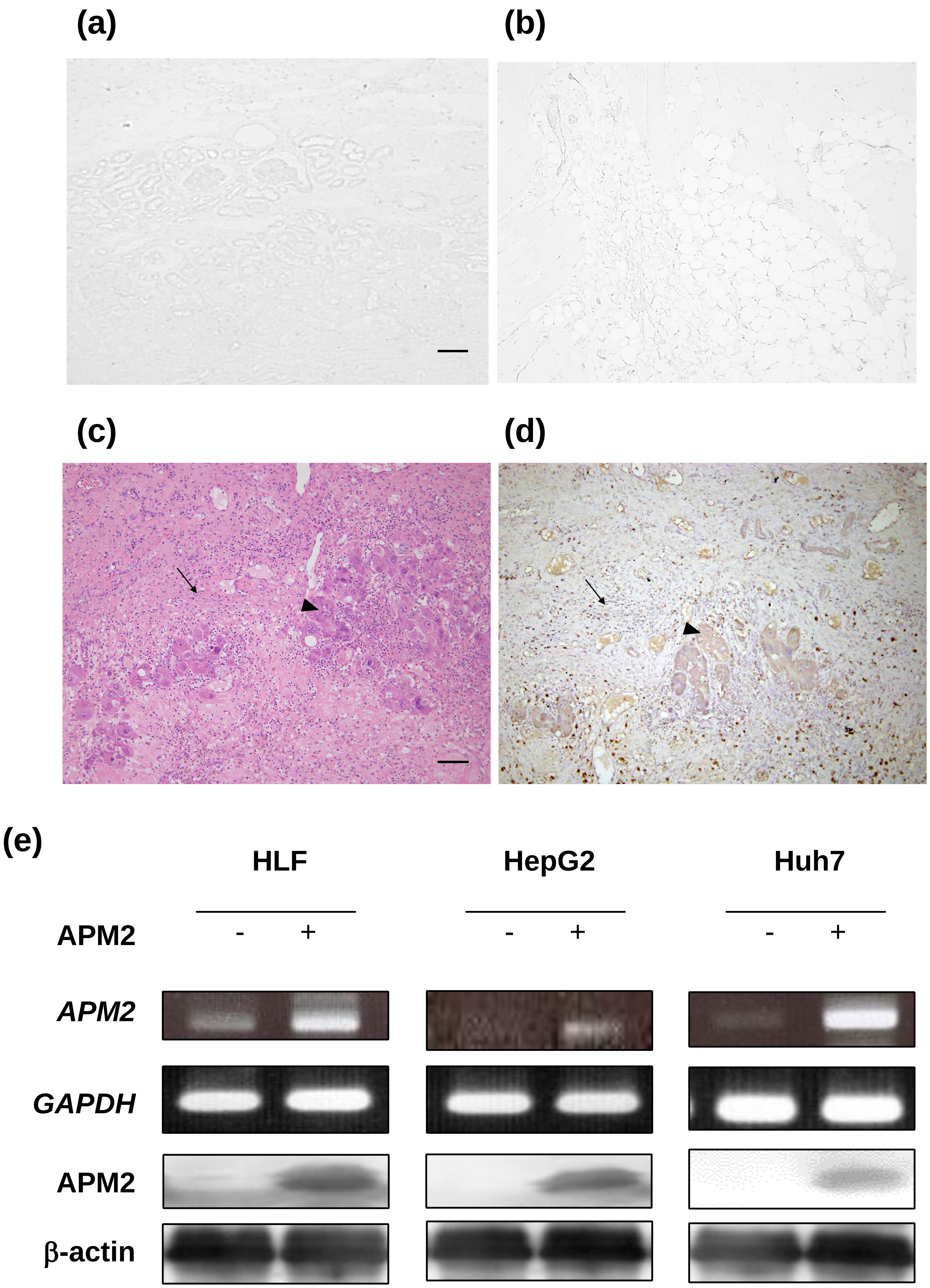

Supplementary information 1. Gels and Blots for Fig. 2 and Suppl. Fig S1

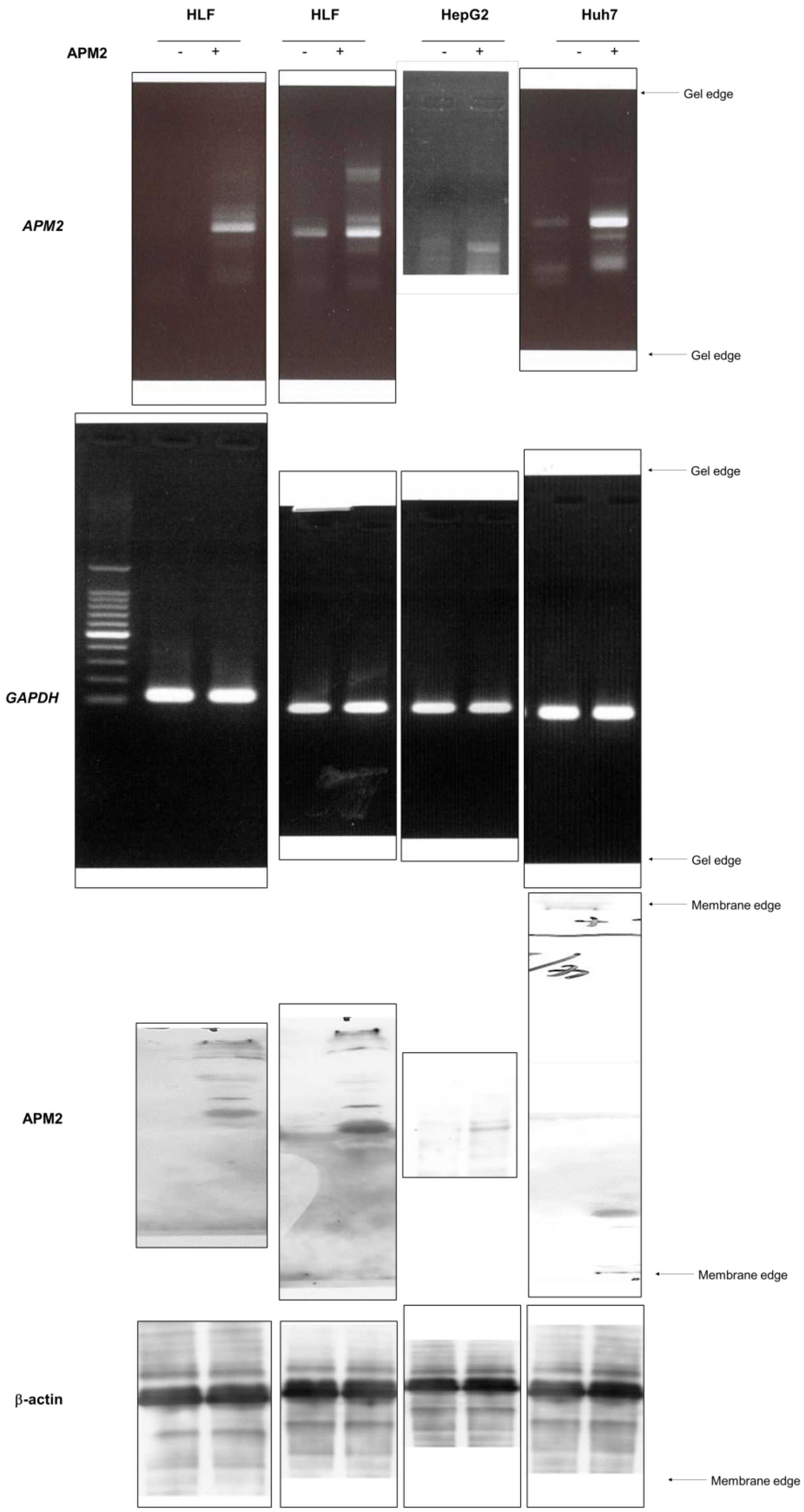

Supplementary information 2. Gels and Blots for Fig. 4

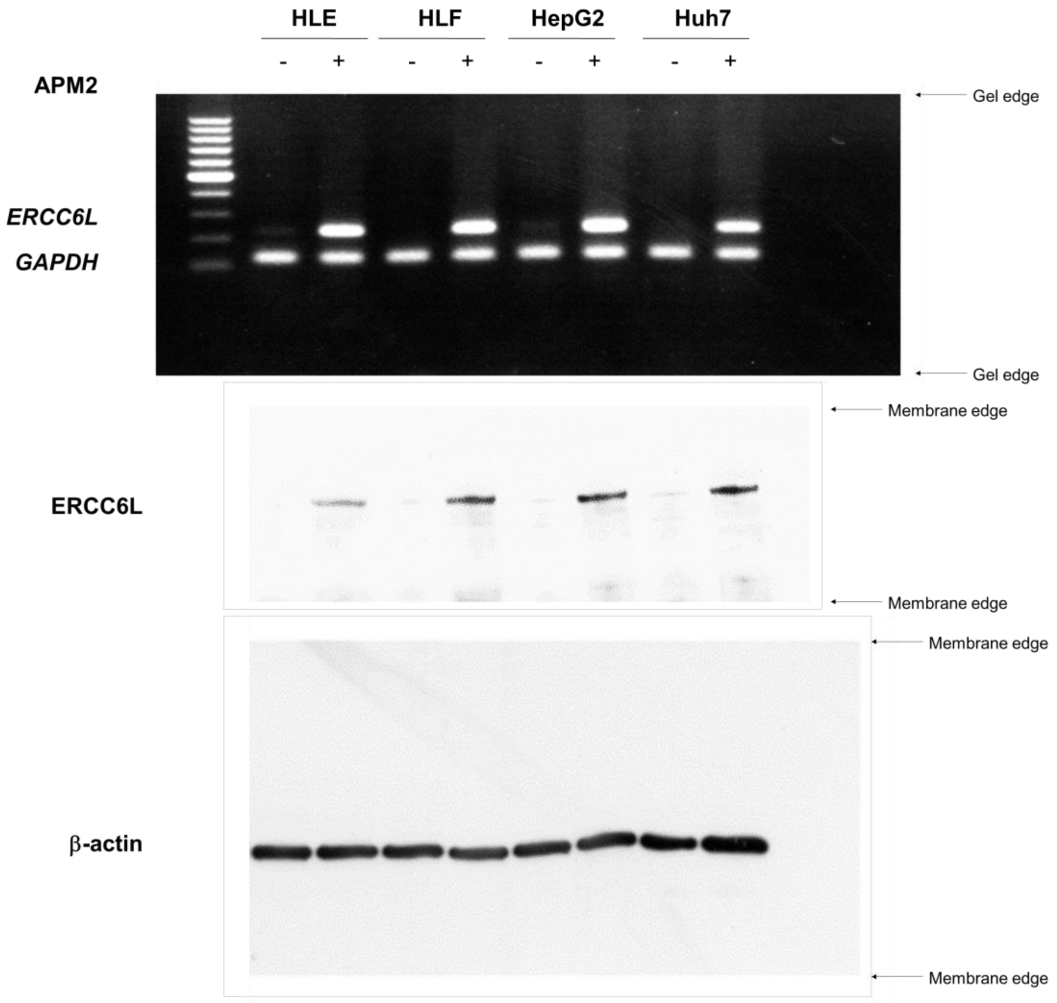

Supplement: Supplementary file 1 — Supplementary Information 1. [file 41598_2021_85498_MOESM1_ESM.pdf]
