# Supplementary material for: Adipose most abundant 2 protein is a predictive marker for cisplatin sensitivity in cancers
Source: Sci Rep. 2021 Mar 18;11:6255. doi: 10.1038/s41598-021-85498-7 (PMC7973578; doi:10.1038/s41598-021-85498-7)
Supplement: Supplementary file 2 — Supplementary Figure S1 Legend. [file 41598_2021_85498_MOESM2_ESM.docx]

**Supplementary Fig. S1**

(a, b) The negative control for the immunohistochemical staining of APM2. The primary antibody was omitted in the immunohistochemical staining to stain the serial sections of normal skin and kidney tissue, which are APM2 positive (Fig. 1e and Fig. 1f). The scale bar represents 100 µm. (c, d) Representative HE and immunohistochemical staining of APM2 in the liver tissues consisting of normal (black arrows) and HCC tissues (black arrowheads). The scale bar represents 100 µm. (e) Representative RT-PCR of *APM2* and *GAPDH* and Western blotting of APM2 and β-actin in APM2 overexpressing HLF, HepG2, and Huh7 cells. The images were grouped from the different gels and separated with the spaces.
